# Genome-Wide Characterization of Alfin-like Genes in *Brassica napus* and Functional Analyses of *BnaAL02* and *BnaAL28* in Response to Nitrogen and Phosphorus Deficiency

**DOI:** 10.3390/plants13172493

**Published:** 2024-09-05

**Authors:** Zexuan Wu, Shiying Liu, Xinyun Zhang, Xingzhi Qian, Zhuo Chen, Huiyan Zhao, Huafang Wan, Nengwen Yin, Jiana Li, Cunmin Qu, Hai Du

**Affiliations:** 1 Integrative Science Center of Germplasm Creation in Western China (CHONGQING) Science City, College of Agronomy and Biotechnology, Southwest University, Chongqing 400715, China; w1045123799@126.com (Z.W.); liushiying08@163.com (S.L.); 18624170225@163.com (X.Z.); qxz799252@163.com (X.Q.); chenzhuo0405@163.com (Z.C.); zhaohuiyan@swu.edu.cn (H.Z.); wanhua05@163.com (H.W.); nwyin80@swu.edu.cn (N.Y.); ljn1950@swu.edu.cn (J.L.); drqucunmin@swu.edu.cn (C.Q.); 2 Academy of Agricultural Sciences, Southwest University, Chongqing 400715, China; 3 Engineering Research Center of South Upland Agriculture, Ministry of Education, Chongqing 400715, China

**Keywords:** *Brassica napus* L., Alfin-like gene family, expression analysis, gene function, nitrogen, phosphorus

## Abstract

Alfin-like proteins (ALs) form a plant-specific transcription factor (TF) gene family involved in the regulation of plant growth and development, and abiotic stress response. In this study, 30 ALs were identified in *Brassica napus* ecotype ‘Zhongshuang 11’ genome (BnaALs), and unevenly distributed on 15 chromosomes. Structural characteristic analysis showed that all of the BnaALs contained two highly conserved domains: the N terminal DUF3594 domain and the C-terminal PHD-finger domain. The BnaALs were classified into four groups (Group I-IV), supported by conserved intron–exon and protein motif structures in each group. The allopolyploid event between *B. oleracea* and *B. rapa* ancestors and the small-scale duplication events in *B. napus* both contributed to the large *BnaALs* expansion. The promoter regions of *BnaALs* contained multiple abiotic stress *cis*-elements. The *BnaALs* in I-IV groups were mainly expressed in cotyledon, petal, root, silique, and seed tissues, and the duplicated gene pairs shared highly similar expression patterns. RNA-seq and RT-qPCR analysis showed that *BnaALs* were obviously induced by low nitrogen (LN) and low phosphorus (LP) treatments in roots. Overexpressing *BnaAL02* and *BnaAL28* in *Arabidopsis* demonstrated their functions in response to LN and LP stresses. *BnaAL28* enhanced primary roots’ (PRs) length and lateral roots’ (LRs) number under LP and LN conditions, where *BnaAL02* can inhibit LR numbers under the two conditions. They can promote root hair (RH) elongation under LP conditions; however, they suppressed RH elongation under LN conditions. Our result provides new insight into the functional dissection of this family in response to nutrient stresses in plants.

## 1. Introduction

The Alfin-like (AL) gene family is one of the plant-specific transcription factor (TF) gene families, which is characterized by an N-terminal highly conserved DUF3594 domain, a C-terminal conserved PHD-finger domain (Cys4HisCys3-type), and a variable V domain between them [1]. The N-terminal DUF3594 domain is involved in protein interaction and in the binding feature to the conserved *cis*-element GNGGTG/GTGGNG to regulate the downstream target gene expression [2,3,4]. The C-terminal PHD finger domain functions in nucleoproteins and participates in nuclear localization [4,5], and can combine with active histones to modify chromatin to regulate plant stress resistance and other processes [3].

Since the first *Alfin1* gene was isolated from salt-tolerant alfalfa cells, many homologous genes have been cloned and functionally validated in plants [1,2,5,6,7]. In *Arabidopsis*, overexpression of *AtAL5* gene improved plant tolerance to cold, drought and salt stresses [2], while *AtAL*6 and *AtAL*7 genes are involved in regulating seed germination, root development and stress resistance [8,9]. Moreover, the *AtAL*6 gene also plays an important role in root hair (RH) elongation under low-phosphorus (LP) conditions [9]. Similarly, the *Atriplex hortensis AhAL1* gene [1], *Medicago sativa MsAL1* gene [3], *Glycine max GmPHD2* gene [4], and *Solanum lycopersicum SlAL3* gene have been proved to improve salt and drought tolerance [10], as well as promote root development. These studies indicate that AL genes play prominent roles in plant stress resistance, growth and development. Accordingly, the AL gene family has been systematically identified and analyzed in many plant genomes, including *Arabidopsis* [11], *Brassica rapa* [12], *Zea mays* [13], *Solanum lycopersicum* [10], *Populus trichocarpa* [14], and *Pyrus bretschenedri* [15], etc. For instance, 7 and 15 AL gene family members were identified in *Arabidopsis* and *Brassica rapa* genomes, respectively [12].

*Brassica napus* L. is a major cultivated oil crop and edible vegetable oil source worldwide. Although the molecular function and genome-wide studies of AL genes have made great progress in plants, there are few reports in *B*. *napus*. A systematic investigation of the evolution and biological function of AL family members is of great significance to further elucidate the evolutionary mechanism and function characteristics of AL genes in *B*. *napus*, especially their roles in development and stress resistance.

In the present study, we conducted global analysis of the AL gene family in *B*. *napus* ‘Zhongshuang 11’ ecotype (ZS11, http://cbi.hzau.edu.cn/bnapus/, accessed on 1 September 2022) genome at the genome-wide level [16], accompanied by a series of bioinformatics analysis of the candidate genes, including gene structural characteristics, phylogenetic relationship, conserved amino acid motif, collinearity relationship, *cis*-elements in the promoter region, and its upstream regulating network. Then, we analyzed the spatiotemporal expression profiles of candidates in 59 ZS11 tissues/organs at different developmental stages. RNA-Seq and RT-qPCR were used to analyze the expression patterns of the candidate genes under low-nitrogen (LN) and LP stresses in the roots of ZS11, respectively. Functionally, *BnaAL02* and *BnaAL28* gene were overexpressed in *Arabidopsis* and their functions in LN and LP deficiency in roots were analyzed. This study laid the foundation for further function assays of the AL gene family in *B. napus*, and provides new insight into the functional dissection of the family gene in LN and LP stresses’ responses in plants.

## 2. Results

### 2.1. Identification and Phylogenetic Analysis of BnaALs in B. napus Genome

To identify the ALs in *B. napus* genome, we performed a BLASTP search in the BnPIR database of *B. napus* ecotype ‘Zhongshuang 11’ (ZS11), using the protein sequences of *Arabidopsis* AL proteins (AtALs) from TAIR (http://www.arabidopsis.org/, accessed on 1 September 2022) as queries. After discarding the redundant and severely missing sequences, the remaining were further examined to ensure each possesses the typical domains of this gene family using SMART (http://smart.embl-heidelberg.de/, accessed on 2 September 2022) and PFAM (http://pfam. xfam.org/, accessed on 2 September 2022) online software. Finally, we obtained a total of 30 typical AL gene family members from ‘ZS11’ genome (*BnaALs*), which were named from *BnaAL01* to *BnaAL30*, according to their chromosomal locations. We identified 15 and 12 non-redundant ALs in *B. rapa* (*BrALs*) and *B. oleracea* (*BoALs*) genomes, respectively, by the same methods (Table S1). Physicochemical property analysis showed that the length of the BnaALs ranged from 210 to 269 amino acids. The molecular weight of the BnaALs ranged from 23.38 to 30.69 kDa, and the isoelectric points were concentrated between 4.79 and 5.72. Subcellular localization analysis showed that all BnaALs are present in the nucleus, consisting of the functional characteristics of typical TFs.

**Figure 1:**
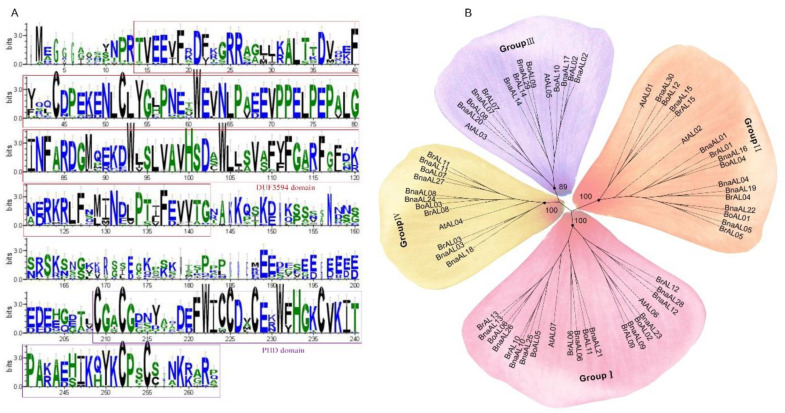
Conserved structural diagram and phylogenetic tree of 64 AL genes. (**A**) The sequence features of the 30 BnaZSALs. (**B**) The phylogenetic tree of 7, 15, 12, 30 AL proteins from *Arabidopsis* (At), *B. rapa* (Br), *B. oleracea* (Bo) and *B. napus* (Bn) based on multiple-sequence alignment with 1000 bootstrap replicates. The inner circle is marked in purple, blue, pink and yellow, representing Groups I-IV, and the bootstrap value for each group is shown.

Multiple-sequence alignment analysis showed that the N-terminal of all BnaALs contained the typical conserved DUF3594 domain, with an average of ~128 amino acids; the C-terminal contained the conserved PHD finger domain, with an average of ~50 amino acids (Figure 1A). The sequence similarity among these two domains was greater than 80%, indicating high sequence conservation, while the conservation of the sequence between them was relatively low. To determine the evolutionary relationship of BnaALs, we constructed a neighbor-joining (NJ) phylogenetic tree based on multi-sequence alignment of the 64 full-length AL protein sequences from *B. napus* (30), *B. rapa* (15), *B. oleracea* (12), and *Arabidopsis* (7). On the basis of the topology and bootstrap values of the tree, these proteins were classified into four groups: Groups I–IV (Figure 1B). Among them, Group I was the largest, including ten BnaALs (33%), three BoALs, four BrALs, and two AtALs; Group II consisted of eight BnaALs (27%), three BoALs, four BrALs, and two AtALs; Group III had six BnaALs (20%), three BoALs, three BrALs, and two AtALs; and Group IV contained six BnaALs (20%), two BoALs, three BrALs, and one AtAL (Figure 1B). In the phylogenetic tree, the number of the BnaALs was five times that of the AtALs in Group I; however, it was four times that of the AtALs in Group II and was three times those in Groups III and IV. This suggested that the expansion rate in distinct groups varies, which might reflect the specific functional needs of different groups during evolution.

### 2.2. Gene Structural and Protein Motif Analysis of BnaALs

**Figure 2:**
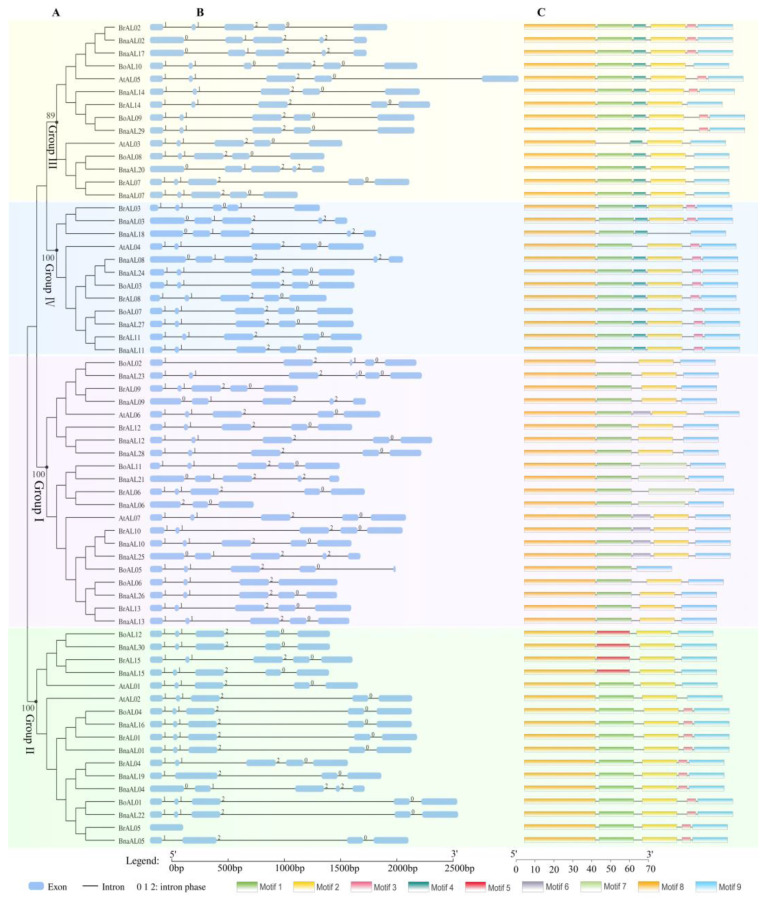
The gene structure and conserved motif analysis of AL gene family. (**A**) The neighbor-joining tree was generated based on the sequence alignment of 64 AL genes from *Arabidopsis*, *B. oleracea*, *B. rapa* and *B. napus*. The different color boxes represent the four groups. (**B**) The gene structures of the candidate genes generated by the Gene Structure Display Server (GSDS 2.0). Blue boxes indicate exons, and black lines indicate introns. Numbers 0, 1 and 2 represent intron phases. (**C**) The conserved motifs of the candidate proteins detected by the MEME and shown as different color boxes using TBtools v1.098667. The sequence information of the motifs was provided in Table S2.

As shown in Figure 2, the structure of *ALs* in *Arabidopsis*, *B. rapa*, *B. oleracea* and *B. napus* is evolutionarily conserved, generally containing five exons separated by four introns. Up to 90% (27) of the 30 *BnaALs* contained four introns, and 91% of them contained three introns in the coding region of the N terminal DUF3594 domain. Among the four groups, the exon–intron structure of Group II was relatively conserved compared to the other three groups, all of which contained four introns. Most *BnaALs* in Groups I, III, and IV also had four introns, excepting for *BnaAL06*, which contained two introns, *BnaAL05* and *BnaAL19*, which contained three introns, and *BnaAL23*, which contained five introns. Moreover, the insertion sites and phases of the introns in most *BnaALs* were highly conserved among the four groups. And the intron insertion patterns were conserved among *BrALs*, *BoALs*, *AtALs*, and *BnaAL* homologous genes, indicating that the intron insertion patterns of *AL* genes were highly conserved across different species.

The MEME online software (https://meme-suite.org/meme/tools/meme, accessed on 2 September 2022) was further applied to identify motifs in the 30 BnaALs, 15 BrALs, 12 BoALs, and 7 AtALs. In all, seven conserved motifs with variable length (6–28 amino acids) were detected in the regions between the N-terminal DUF3594 domain (Motif 8) and the C-terminal PHD finger domain (Motif 9) (Table S2). While Motif 1 and Motif 2 were shared by the four groups, the remaining were only present in members of one or two groups. Motif 3 was present in 11 members of Group II, 7 members of Group III and all members of Group IV except BnaAL18. Motif 4 was highly conserved in Groups III and IV; however, AtAL4 did not contain this Motif. Motif 5 was distributed in four proteins of Group II; Motifs 6 and 7 were distributed among five and four proteins in Group III, respectively. Motif 2 contained a highly conserved ‘EEEEEEEE’ sequence that may be relevant to its function (Table S2).

### 2.3. Allopolyploidy and Small-Scale Duplication Events Contributed to the Large Expansion of BnaALs

**Figure 3:**
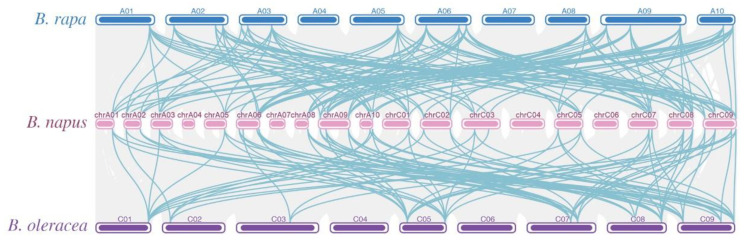
Collinear relationship of the AL family in *B. rapa*, *B. napus*, and *B. oleracea*. The blue, pink, and purple bars represent the chromosomes of Al genes in *B. rapa*, *B. napus* and *B. oleracea*, respectively. The collinear blocks (between the genomes of *B. napus* and *B. rapa*, *B. napus* and *B. oleracea*) were indicated in gray lines.

Consistent with the situation in other plant species [11,12,13,14,15], chromosomal location analysis showed that the 30 *BnaALs* were unevenly distributed in 15 of 19 *B. napus* chromosomes (Figure 3). The chromosomes A06 and C09 possessed the most *BnaALs* (four genes), whereas A01, A03, A05, A08, and C01 only had one each. The *BnaALs* were equally distributed in A_n_ and C_n_ subgenomes, each with 15 genes (Figure 3). Moreover, the number of *BnaALs* in A_n_ and C_n_ subgenomes was similar to that of the *AL*s in the ancestors of *B. napus*, where *B. rapa* and *B. oleracea* had 15 and 12 *AL*s, respectively.

Collinear relationship analysis showed that the 30 *BnaALs* had a collinear relationship with *B. rapa* and/or *B. oleracea* homologs (Table S3). Except for *BnaAL07*, which did not have a genetic background with *B. rapa*, the remaining 29 *BnaALs* had both a *B. rapa* and *B. oleracea* genetic background. Among the 30 *BnaALs*, 10 genes (33%) were derived from the two ancestor genomes, while 20 genes were from small-scale duplication events within *B. napus*, including ten (33%) from homologous exchange (HE) events, one (3%) from segmental exchange (SE) events, and nine (30%) from segmental duplication (SD) events. The *BnaALs* from HE events were all located in the C_n_ subgenome. No tandem duplication (TD) events were detected in *BnaALs*. These results indicated that both allopolyploidy and small-scale duplication events were the main amplification modes of *BnaALs*. And the *B. napus* genome tended to retain the *ALs* from *B. rapa* and then replaced the orthologous genes from *B. oleracea* in the C_n_ subgenome through HE events.

### 2.4. The Expression of BnaALs Might Be Regulated by Multiple Factors

**Figure 4:**
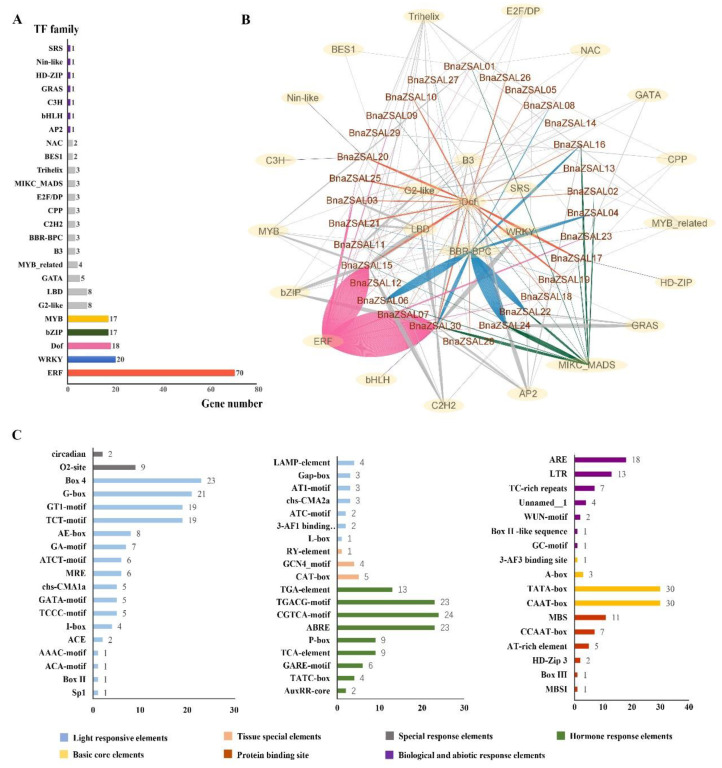
Regulation mechanism analysis in the promotor regions of *BnaALs*. (**A**) The TF gene families with potential binding sites in the promoter regions of the 30 *BnaAls*. (**B**) The potential TF binding network of the *BnaALs* predicted by the PlantTFDB tool. (**C**) The *cis*-elements in the promoter regions of the *BnaALs*. The abscissa represents the number of *BnaALs*.

The −1500 bp promoter sequences of the 30 *BnaALs* were used to predict the potential transcriptional regulators though PlantTFDB database. In all, 199 potential TF binding sites were identified, belonging to 25 TF gene families (Figure 4). The most abundant TFs belonged to the ERF (70 genes), WRKY (20 genes), and Dof (18 genes) families (Figure 4A). There were 70 ERF, 20 WRKY, and 17 bZIP TFs that might bind to 12, 4, and 6 *BnaALs*, respectively, suggesting some TFs might commonly regulate the same target gene (Figure 4B). For instance, many candidate ERF TFs had potential binding sites in both *BnaAL15* and *BnaAL30* promoters (Table S4). Similarly, *BnaAL06*, *BnaAL19* and *BnaAL22* might be the common targets of many BBR-BPC TFs (Figure 4B). Meanwhile, some TFs may have muti-target genes (Figure 4B). For example, the three candidate MIKC_MADS TFs may bind to the promoters of 13 *BnaALs*, the three Trihelix TFs may bind to 11 *BnaAL*s promoters, and the 3 BBR-BPC TFs may regulate 12 *BnaALs* (Figure 4B). The Dof, MIKC_MADS, BBR-BPC and ERF families may regulate the most *BnaALs* (Figure 4B), suggesting their important roles in regulating the expressions of *BnaALs*. The remaining TF families (e.g., LBD, G2-like, GATA, etc) appeared to regulate only a few *BnaALs*.

PlantCARE online software (https://bioinformatics.psb.ugent.be/webtools/plantcare/html/, accessed on 2 September 2022) analyses identified 1929 *cis*-acting elements in the promoters of the 30 *BnaAL*s, which were classified into 55 types (Figure 4C and Table S5). Many hormone response *cis*-elements, stress response *cis*-elements and TF binding sites were obtained. The hormone response *cis*-elements clustered into nine types, including TATC-box, TCA-box, and P-box, etc. (Figure 4C), indicating these *BnaALs* may be regulated by MeJA, ABA, IAA, etc. The stress response *cis*-elements were classified into six types, such as AREs, LTRs, and WUN-motif (Figure 4C), which may be involved in the response to biotic and abiotic stresses, such as anaerobic, low temperature, etc. The TF binding sites mainly included MYB binding sites (MBS element), MYBHv1 binding sites (CCAAT-box), and ATBP-1 binding sites (AT-rich). These results suggest that the expression of *BnaALs* may be regulated by multiple factors.

### 2.5. BnaALs Exhibited a Wide Spatiotemporal Expression Profiles in B. napus

**Figure 5:**
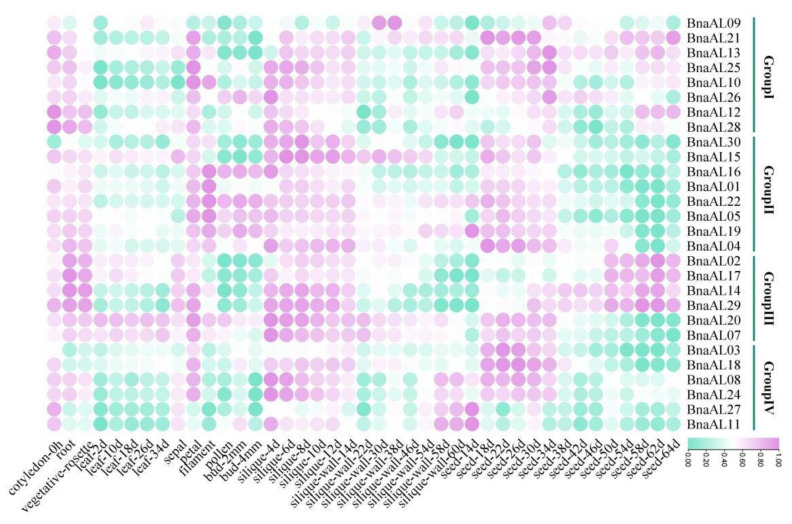
Spatiotemporal expression profile of *BnaALs* in different tissues during *B. napus* development. The expression data of the 30 *BnaALs* in 59 *B. napus* tissues/organs across different development stages were obtained from the BnTIR database (http://yanglab.hzau.edu.cn). In the color bar, purple represents a high level of expression, and green represents little or no expression. “mm” represents the germinating length; “h” and “d” indicate hour and day.

We inspected the spatiotemporal expression patterns of the 30 *BnaALs* in 59 *B. napus* ecotype ‘ZS11’ tissues/organs across different development stages based on the BnTIR database (http://yanglab.hzau.edu.cn, accessed on 1 June 2023). Twenty-eight *BnaALs* had detectable expression levels (FPKM ≥ 1), except *BnaAL06* and *BnaAL23*, which had no detectable transcript levels (Figure 5). Overall, the majority *BnaALs* in Group I-IV were preferentially highly expressed in root, cotyledon, rosette, petal, silique, and seed tissues. Moreover, the homologs generally had a conserved expression pattern in the same group or at least in the same clade, indicating their conserved and redundant functions. In Groups I and II, almost all of the 16 genes shared conserved expression patterns (except for *BnaAL09*) and were partial to the early development stages of silique and seed tissues. In Group III, *BnaAL02*, *BnaAL14*, *BnaAL17*, and *BnaAL29* genes were highly expressed at the later development stage of seeds, while the last two genes (*BnaAL07* and *BnaAL20*) had a conserved and preferential expression levels at the early development stages of seeds. In Group IV, *BnaAL03*, *BnaAL08*, *BnaAL18*, and *BnaAL24* were preferentially highly expressed in the early development stages of seeds, whereas *BnaAL11* and *BnaAL27* were highly expressed in the later development stage of siliques. To explore the fates of the duplicated *BnaALs*, we calculated the sequence identity in the two conserved domains (DUF3594 and PHD domains), full-length protein sequence, coding sequence (CDS) and the promoter (−1500 bp) sequences of the 15 duplicated gene pairs based on collinearity analysis, respectively (Table S6). The results showed that the average identity of the full-length CDS and protein sequences of the duplicated gene pairs was ~95%, while it was ~59% in the promoter regions. Correlation analysis (Pearson’s correlation coefficient) showed that the expression profiles of the duplicated gene pairs were all >0.60 (Table S6), suggesting their functional redundancy. These results proved that the duplicated *BnaALs* shared a conserved sequence feature and expression profile, which may be attributed to their relative short origin time in *B. napus*.

### 2.6. BnaALs Were Inducible under LN and LP Conditions by RNA-seq and RT-qPCR Assay

**Figure 6:**
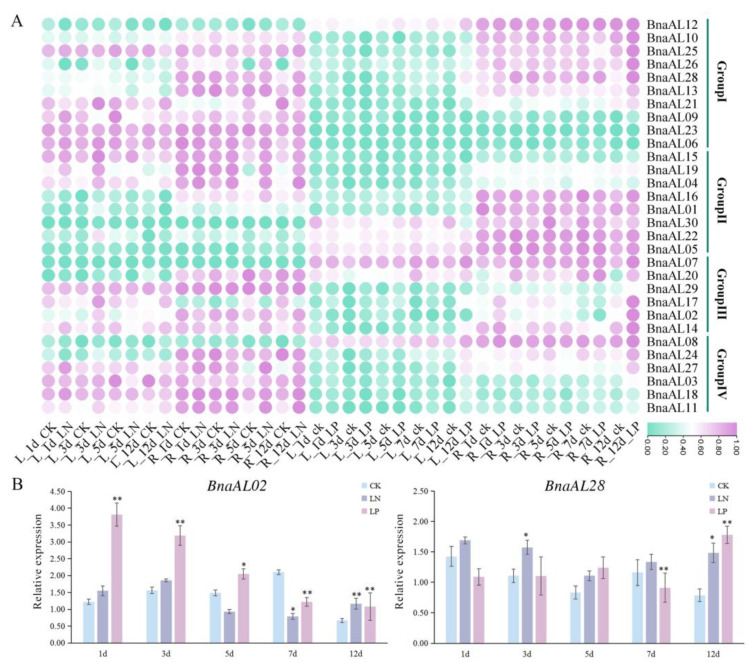
Expression pattern of *BnaALs* under low-nitrogen (LN) and low-phosphorous (LP) treatments. (**A**) Expression pattern analysis of the 30 *BnaALs* under LN and LP treatments based on RNA-seq datasets (BnaGADB, http://www.bnagadb.cn/). “L” = leaf; “R” = root; “CK” represents control treatment (0 h). “1 d”, “3 d”, “5 d”, and “12 d” represent the days after LN and LP treatments. (**B**) Relative expression pattern analysis of *BnaALs* under LN and LP treatments in *B. napus* by RT-qPCR method. “*” and “**” mean significant difference at the 0.05 and 0.01 probability levels, respectively.

To explore the potential functions of *BnaALs* in nutrient utilization in *B. napus*, the LN and LP stress RNA-seq dataset (http://www.bnagadb.cn/, accessed on 15 October 2023) constructed in our lab was used in this study. The results showed that the majority *BnaALs* were preferentially expressed in ZS11 roots compared to leaves (Figure 6A). Moreover, the *BnaAL*s in Groups I-IV were generally induced by LN and LP stresses in roots. Under LN conditions, the expression levels of *BnaALs* in Groups II-IV were generally decreased in roots after 1d treatment, and then increased at the late stages (5 and 12d). In leaves, the expressions of most *BnaALs* in Groups I, III, and IV were up-regulated after 1 and 3d treatments. Under LP conditions, the expressions of *BnaALs* in Groups I and III were significantly up-regulated after 1 and 3d treatments, were down-regulated after 5d treatments, and then were significantly increased after 12d treatments. The expressions of *BnaALs* in Groups II and IV were generally down-regulated after 1, 3, 5 and 7d treatments in roots, and then were significantly up-regulated after 12d. The *BnaALs* were not obviously induced in the leaves under LP treatments.

To further confirm the LN- and LP-induced expression profiles of *BnaALs* in *B. napus* seedling roots, *BnaAL02* and *BnaAL28* with obvious LN- and LP-induced expressions from Groups I and III were selected for reverse transcription–quantitative polymerase chain reaction (RT-qPCR) assay, respectively. The expression profiles of the two genes were similar to those of the RNA-seq analyses (Figure 6B). For instance, under LN treatment, the expression of *BnaAL02* was significantly down-regulated at 7d, whereas *BnaAL28* was significantly up-regulated at 3 and 12d. Under LP treatment, the expression of *BnaAL02* was significantly up-regulated at all time points, and the expression of *BnaAL28* was significantly down- and up-regulated at 7d and 12d, respectively. These results confirmed that the two genes may play a role in LN and LP response in *B. napus*.

### 2.7. BnaALs Regulated Multiple LN- and LP-Induced Roots Phenotypes in Arabidopsis

To confirm the functions of *BnaAL02* and *BnaAL28* in LN and LP stress response, we constitutively expressed them in *Arabidopsis* under the driving of CaMV35S promoter (*35Sp::BnaAL02* and *35Sp::BnaAL28*). For LN and LP stress assay, the wild-type (Col0), the *al6* mutant (the homolog of *BnaAL28* in *Arabidopsis*), and the overexpressed (OE) *BnaAL02* and *BnaAL28* transgenic *Arabidopsis* lines were cultured in normal Hogland’s solid medium for 3d and then transferred to LN and LP solid media for 7d.

**Figure 7:**
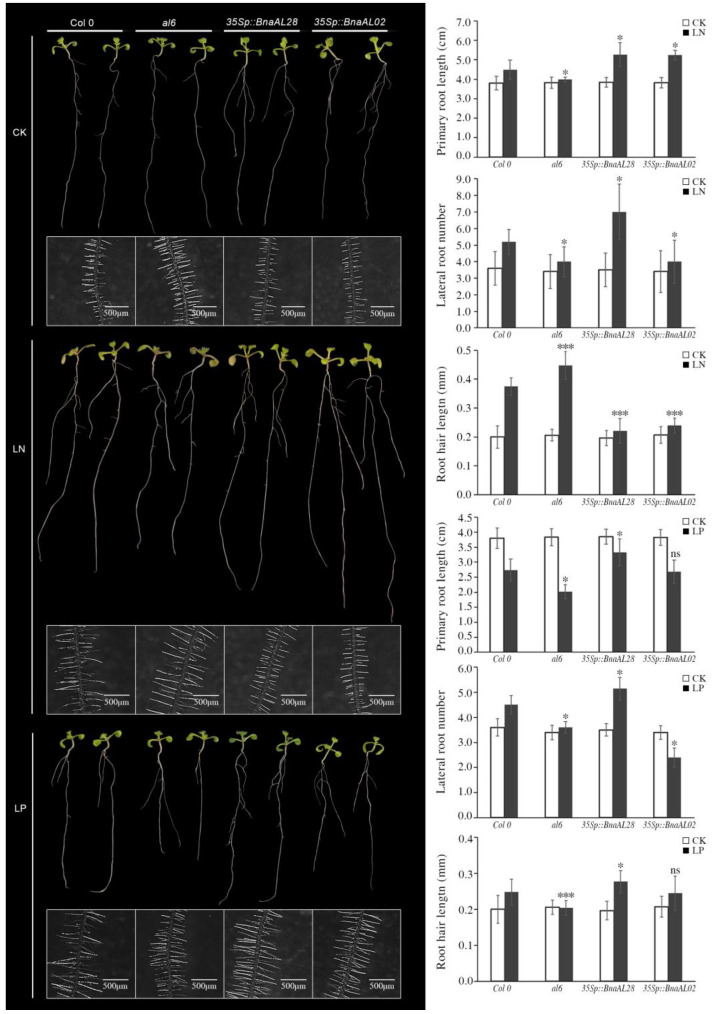
Phenotype analyses of the overexpression lines of *BnaAL02* and *BnaAL28* under LN and LP treatments in *Arabidopsis*. Comparison of primary root length, total number of lateral roots, and root hair length per plant of the Col0, *al6*, *35Sp::BnaAL02* and *35Sp::BnaAL28 Arabidopsis* seedlings were performed by growing on the control, LN, and LP media, respectively. “*”, “**” and “***” mean significant difference at the 0.05, 0.01 and 0.001 probability levels, respectively.

Under control condition, the primary root (PR) length, number of lateral roots (LRs), and root hairs (RHs) length of Col0, *al6*, *35Sp::BnaAL02* and *35Sp::BnaAL28* lines were similar (Figure 7). However, compared to Col0, the PR length and the LR number significantly decreased in the *al6* mutant under LN conditions, while were significantly increased in *35Sp::BnaAL28* lines. On the contrary, a significant increase in the RH length was observed in the *al6* mutant, while that in the *35Sp::BnaAL28* lines was significant decreased. Similarly, under LP condition, a significant decrease in the PR length, the LR number and the RH length was observed in the *al6* mutant compared to Col0, while those of the *35Sp::BnaAL28* lines were significant increased. These results suggested that *BnaAL28* can increase the RH length under LP conditions and have the opposite effect in LN conditions, and can increase PR length and the LR numbers under LP and LN conditions. For, *BnaAL02* gene, under LN conditions, a significant increase in the PR length and the RH length was observed in *35Sp::BnaAL02* lines, while the LR number was significantly decreased compared to Col0. Under LP conditions, the length of the PR and the RH of *35Sp::BnaAL02* lines was similar to that of the Col0, while the LR numbers were significantly decreased. These results suggested that *BnaAL02* can promote the PR length and inhibit the RH length under LN conditions, and can inhibit the LR number under LP conditions.

## 3. Discussion

Alfin-like TF genes are widely present in plant genomes and play important roles in plant response to abiotic stress and growth and development [17]. To date, the Alfin-like family has been systematically identified and analyzed in many plant genomes, such as *Arabidopsis* (7 *Als*) [11], *B. rapa* (12 *Als*) [12], *Pyrus Bretschenedri* (9 *Als*) [15], *Populus trichocarpa* (9 *Als*) [14], and *Solanum lycopersicum* (11 *Als*) [18], which has proven it is a plant-specific gene family. In this study, a total of 30 Alfin-like family members were identified from the genome of *B. napus* ecotype ‘ZS11’, which is significantly greater than that in the reported species [8,11,12,13,14]. This indicates that the gene family has been extensively amplified in *B. napus*. It is known that *B. napus* is an allotetraploid (A_n_A_n_C_n_C_n_) evolved by a spontaneous hybridization event between *B. rapa* (A_n_A_n_) and *B. oleracea* (C_n_C_n_) about 7500 years ago [19], and cruciferous plants have collectively experienced a whole-genome triplication (WGT) event during the evolution. As a result, it was expected that the seven *Arabidopsis ALs* might be expanded to ~20 genes in *B. oleracea* or *B. rapa*, and ~40 in *B. napus* genomes. However, in this study, only 12, 15, and 30 *ALs* were identified in these three species, respectively, indicating that 40% of *BoALs*, 25% of *BrALs* and 25% of *BnaALs* were lost during evolution and relatively more *ALs* derived from *B. rapa* were reserved in *B. napus* after the allopolyploid event. In addition, we found that the number of *BnaALs* in the A_n_ and C_n_ sub-genomes of *B. napus* was basically the same as its *B. rapa* and *B. oleracea* ancestors. Moreover, many *BnaALs* showed a collinearity relationship with their homologs in *B. rapa* or *B. oleracea*, suggesting that the *BnaALs* were mainly derived from the allopolyploid event. Furthermore, consistent with the findings in other species [8,11], our results proved that the chromosome HE and SD events were the main amplification forces of the *BnaALs* in *B. napus* as well. Together, a genome-wide duplication event (such as allopolyploid and and WGT events) and a small-scale duplication event (such as HE and SD events) are the major driving forces for *Als’* expansion in plants.

Many studies have proven that members of the Alfin-like family are widely involved in drought, high salt, and cold stress processes in plants [1,6,10,12]. For example, 11 *BrALs* genes in *B. rapa*, 6 *GmPHDs* in soybean, and 2 *SlALs* in tomato are universally induced by drought, cold, and salt stresses [6,10,12]. In *Arabidopsis*, *AL5* and *AL6* genes were demonstrated to play a role in salt and drought tolerance, while *AL5* can enhance freezing tolerance [2]. In this study, *cis*-element analysis showed that 11 *BnaALs* contained drought inducibility response *cis*-elements, 13 *BnaALs* contained low-temperature responsiveness *cis*-elements, and 23 *BnaALs* contained ABA responsiveness *cis*-elements in the promoter regions of the 30 *BnaALs* (Figure 4), suggesting their potential roles in abiotic stress in *B. napus*. In addition, *Arabidopsis AL6* gene was proven to promote RH elongation under P deficiency [9], indicating that *ALs* are involved in nutrient stress response processes as well. Accordingly, in the present study, RNA-seq data showed that *BnaALs* were generally expressed in *B. napus* roots, and many of them were significantly induced by LN and LP treatments in the roots (Figure 6). Consistent with the previous study [9], our results demonstrated that the homolog of *Arabidopsis AL6*, *BnaAL28* could promote the RH elongation under LP condition. Moreover, *BnaAL28* can effectively promote the PR extension and LR development under LP conditions, whereas it can promote the number of LRs and the elongation of PR under LN conditions. This indicated a wide role of *BnaAL28* in nutrient stress response processes in *B. napus*. Meanwhile, we demonstrated that *BnaAL02* in Group III can inhibit the roots’ development under LP and/or LN conditions. But the functional features of these two genes were not the same, suggesting the functional divergent trend of the homologous genes during the evolution. In addition, some *Als* in rice and *Arabidopsis* were reported to play important roles in plant growth and development, such as regulating root development, seed shape, and seed germination, etc. [17]. In this study, our expression analyses revealed that all of the 30 *BnaALs* exhibited a typical temporal and spatial expression profile in *B. napus* across different developmental stages, with most *BnaALs* being preferentially expressed in root, cotyledon, rosette, petal, silique, and seed tissues (Figure 5). This supplied important information regarding gene functions of the AL gene family in the growth and development in *B. napus*.

In conclusion, our results lay a solid foundation for future studies on the evolution and biological functions of the Alfin-like family, and contribute to the long-term goal of improving LN and LP stresses in *B. napus*.

## 4. Materials and Methods

### 4.1. Identification of AL Genes in B. napus and Other Plants

The AtALs were obtained from the TAIR website (http://www.arabidopsis.org/, accessed on 1 September 2022). To identify the AL-encoding genes in *B. napus* genome, we performed a BLASTP search in the BnPIR database (http://cbi.hzau.edu.cn/bnapus/index.php, accessed on 1 September 2022) [20], using the known AtALs as queries with a low-stringency criterion (expectation value < 1.0). After deleting the redundant sequences, the remaining sequences were examined by SMART (http://smart.embl-heidelberg.de/, accessed on 2 September 2022) and PFAM tools (http://pfam.xfam.org/, accessed on 2 September 2022) to ensure the candidate protein sequences had the typical domains and sequence characteristics of this gene family. Using the same method, we also identified the BrALs (*Brassica rapa*) and BoALs (*Brassica oleracea*) from the Phytozome v12.1 database (http://www.Phytozome.net, accessed on 3 September 2022) [21], respectively. The biochemical properties of the candidate proteins were determined using the ExPaSy tool (https://web.expasy.org/compute_pi/, accessed on 3 September 2022) [22], and the subcellular localization was investigated using Plant-mPLoc (http://www.csbio.sjtu.edu.cn/bioinf/plant-multi/, accessed on 4 September 2022).

### 4.2. Phylogenetic Analysis of AL Family in B. napus

To explore the evolutionary relationship of this gene family in *B. napus*, *B. oleracea*, *B. rapa*, and *Arabidopsis*, we performed multiple-sequence alignment of the obtained protein sequences of BnaALs, BrALs, BoALs, and AtALs using the MAFFT version 7 with default parameters (https://mafft.cbrc.jp/alignment/server/, accessed on 4 September 2022). Then, the alignment was visualized with Weblogo [23]. Subsequently, a phylogenetic tree was built using MEGA v7.0 with the neighbor-joining (NJ) method based on the multiple-sequence alignment [24]. The parameters used in the phylogenetic analyses were as follows: Poisson correction, bootstrap with 1000 replicates, and pairwise deletion. Finally, iTOL online software (https://itol.embl.de/itol.cgi, accessed on 7 September 2022) was applied to view and edit the tree file.

### 4.3. Gene Structure Analysis of BnaALs

The gene structures of *BnaALs*, *BrALs*, *BoALs* and *AtALs* were analyzed using Gene Structure Display Server (GSDS) 2.0 (http://gsds.cbi.pku.edu.cn/, accessed on 1 October 2022) with the DNA and coding sequences [25]. MEME online analysis software (https://meme-suite.org/meme/, accessed on 10 December 2022) was used to predict the conserved amino acid motifs of candidate AL proteins and visualize them using TBtools software [26].

### 4.4. Chromosomal Location and Collinearity Analysis of BnaALs

We acquired the information on the chromosome locations of candidate *BnaALs* from the BnPIR database (http://cbi.hzau.edu.cn/bnapus/, accessed on 12 February 2023). Mapchart v2.2 software was used to draw the chromosome map of *BnaALs* [27]. The cross-genome collinearity relationship of *BnaALs*, *BrALs*, *BoALs*, and *AtALs* was calculated and identified using the TBtools software [26]. The duplication events of *BnaALs* were defined based on the collinearity relationship.

### 4.5. TF Binding Network, Cis-Elements Analysis of BnaALs

The network between *BnaALs* and their possible transcriptional regulators was constructed based on the analyses in the PlantTFDB database (http://planttfdb.cbi.pku.edu.cn/prediction.php) with the upstream 1500 bp promoter sequences of *BnaALs*. Only TFs with a threshold *p*-value < 10^6^ were retained for further analysis. Finally, the network was viewed using Cytoscape 3.6.1 software [28]. The potential *cis*-elements in the promoter regions were also predicted using the PlantCARE online software (http://bioinformatics.psb.ugent.be/webtools/plantcare/html/, accessed on 25 February 2023).

### 4.6. Gene Expression Analysis of BnaALs

We used the RNA-seq dataset in BnTIR database (http://yanglab.hzau.edu.cn/BnTIR, accessed on 1 June 2023) (BioProject: PRJNA612634) to detect the temporal and spatial expression patterns of *BnaALs* across different developmental stages of *B. napus* cultivar ‘Zhongshuang 11 (ZS11). Similarly, to explore the nutrient-responsive expression patterns of *BnaALs*, the RNA-seq dataset of ZS11 seedling leaves and roots under LN and LP treatments were obtained from BnaGADB website (http://www.bnagadb.cn/, accessed on 15 October 2023). TBtools was used to draw the expression heatmap of candidate genes. Pearson’s correlation coefficient was calculated based on the expression levels of homologous genes in different tissues/organs in ZS11.

### 4.7. Plant Materials and Growth Conditions

Seeds of ZS11 were obtained from the College of Agriculture and Biotechnology, Southwest University. The seeds were germinated in individual plastic pots filled with vermiculite, grown in an artificial climatic chamber at 25 °C with a 16:8 h photoperiod (day:night), and watered with Hoagland solution every four days. Then, seedlings at the four-leaf stage were changed from soil culture to hydroponic culture with Hoagland’s solution. The solutions were changed every three days. The seedlings at the five-leaf stage were used for LP and LN treatments. The formulae for the adjusted Hoagland’s solution that were used in the LN and LP treatments are shown in Table S7, respectively. For each treatment, three biological replicates were performed, and each replicate contained five plants. In the same environment, we germinated transgenic *Arabidopsis* on 1/2-strength Hoagland medium for three days, and then moved them to the LP or LN medium to treatment seven days for phenotype analyses.

### 4.8. RT-qPCR Analysis of BnaALs under Low-N and Low-Pi Conditions

The EASYspin total RNA Extraction kit (Biomed, Beijing, China) was used to extract the total RNA from each sample. The concentration and quality of the total RNA were tested using gel electrophoresis and a NanoDrop^TM^ 2000 spectrophotometer (Thermo Fisher Scientific, Wilmington, DE, USA) to confirm that the A260/280 ratio remained at 1.8–2.1, and that the A260/230 ratio exceeded 2.0. The RNA sample was treated with DNase I (Promega, Beijing, China), and was then used for cDNA synthesis by reverse transcription in a 20ul reaction system according to the manufacturer’s instructions of the M-MuLV RT kit (Takara Biotechnology, Beijing, China). The primers of *BnaAL02* and *BnaAL28* used in this experiment were designed using Primer Premier 5 software and are listed in Table S8. *BnaActin7* (GenBank accession No. AF024716) and *BnaUBI* (GenBank accession No. NC027770) served as double reference genes. The SYBR-Green PrimeScript RT-PCR Kit (Takara Biotechnology, Beijing, China) was used for real-time PCR analysis using the CFX Connect^TM^ Real-Time System (Bio-Rad, Chongqing, China). Each reaction system consisted of three technical replicates. The thermocycling parameters included initial denaturation at 95 °C for 5 min, followed by 45 cycles of denaturation at 95 °C for 15 s and annealing at 60 °C for 15 s (the annealing temperature of *BnaAL02* and *BnaAL28* was 59 °C). Finally, we obtained the data (mean standard deviation) of all three independent repeated trials and calculated the relative expression of *BnaAL02* and *BnaAL28* using the 2^(−ΔΔCt)^ method. Error bars represent standard errors from three independent repeated trials. Differences in expression levels in *BnaAL02* and *BnaAL28* according to LN and LP treatments were assessed by one-way analysis of variance (* *p* < 0.05; ** *p* < 0.01) using Excel 2010.

## Data Availability

This study did not report any data.

## Supplementary Materials

The following supporting information can be downloaded at: https://www.mdpi.com/article/10.3390/plants13172493/s1. Table S1. Features of the Alfin-like genes from Brassica napus (BnaALs), Brassica oleracea (BoALs), Brassica rapa (BrALs) and Arabidopsis thaliana (AtALs) identified in this study. Table S2. List of the seven putative motifs of BnaALs by MEME. Table S3. Collinearity relationship of *BnaALs*. Table S4. Transcription factor (TF) binding sites in the promoter regions of *BnaALs*. Table S5. *Cis*-element in the promoter regions of *BnaALs*. Table S6. Homology and expression correlation analysis of the 19 duplicated *BnaALs* pairs. Table S7. List of the normal, -Pi and -N Hoagland’s component used in this study. Table S8. List of the primers used for the real-time PCR analysis in this study. Figure S1. Expression of Col0 and *BnaAL02* and *BnaAL28* transgenic *Arabidopsis* lines by qPCR analysis.
