# Supplementary figures and images for: Genome-Wide Characterization of Alfin-like Genes in Brassica napus and Functional Analyses of BnaAL02 and BnaAL28 in Response to Nitrogen and Phosphorus Deficiency
Source: Plants (Basel). 2024 Sep 5;13(17):2493. doi: 10.3390/plants13172493 (PMC11396871; doi:10.3390/plants13172493)

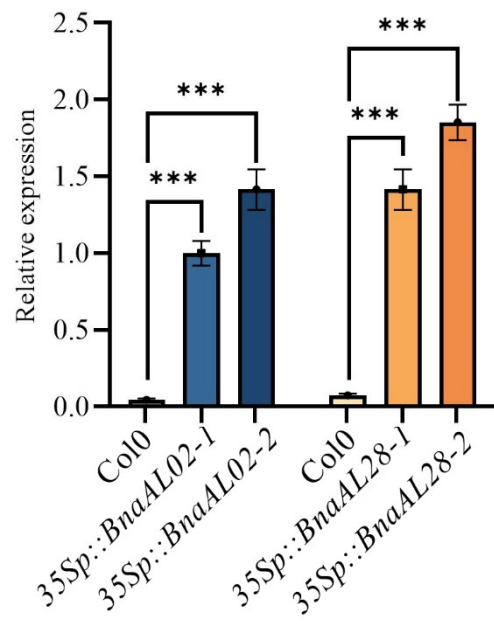

**Figure S1.** Expression of *Col0* and *BnaAL02* and *BnaAL28* transgenic *Arabidopsis* lines by qPCR analysis.

Supplement: Supplementary file 1 [file plants-13-02493-s001.zip › Figur S1.pdf]

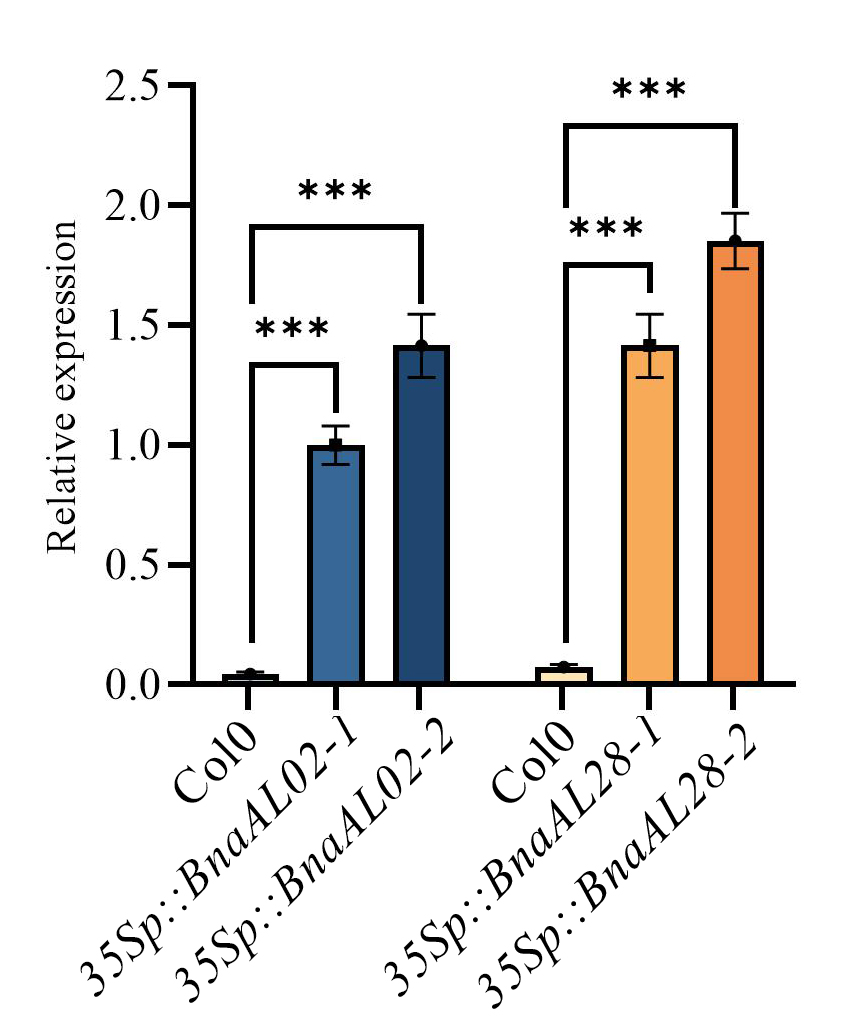

Supplement: Supplementary file 1 [file plants-13-02493-s001.zip › Figure S1.jpg]
